# The Impact of the COVID-19 Pandemic on Medical Training at the Greek National Health Service: A Cross-Sectional Study

**DOI:** 10.3390/epidemiologia6010013

**Published:** 2025-03-06

**Authors:** Ioannis Moutsos, Dimitrios Lamprinos, Evangelia-Georgia Kostaki, Panagiotis Georgakopoulos, Gerasimos Siasos, Evangelos Oikonomou, Kostas A. Papavassiliou, Philippos Orfanos, Georgios Marinos

**Affiliations:** 1 Aberdeen Royal Infirmary, Foresterhill, Aberdeen AB25 2ZN, UK; 2 Department of Family Medicine, Medical School, University of Edinburgh, Argyle House, 3 Lady Lawson Street, Edinburgh EH3 9DR, UK; 3 Department of Hygiene, Epidemiology and Medical Statistics, School of Medicine, National and Kapodistrian University of Athens, 11527 Athens, Greece; ekostakh@med.uoa.gr (E.-G.K.); phorfanos@med.uoa.gr (P.O.); gmarino@med.uoa.gr (G.M.); 4 General Hospital of Athens “G. Gennimatas”, 11527 Athens, Greece; panoskgeorgakopoulos@gmail.com; 5 Third Department of Cardiology, Thoracic Diseases General Hospital Sotiria, University of Athens Medical School, 11527 Athens, Greece; gsiasos@med.uoa.gr (G.S.); boikono@gmail.com (E.O.); 6 First Department of Respiratory Medicine, “Sotiria” Hospital, Medical School, National and Kapodistrian University of Athens, 11527 Athens, Greece; konpapav@med.uoa.gr

**Keywords:** medical training, surgical training, factor analysis, Greece, COVID-19 pandemic

## Abstract

**Introduction:** The COVID-19 pandemic caused significant disruptions to medical training worldwide, particularly for junior doctors, as in-person clinical training was replaced by online education. This study aims to assess the impact of the pandemic on medical training in Greece, focusing on the perceptions of junior doctors across various specialties and exploring the implications for future clinical practice. **Methods:** We conducted a cross-sectional online survey of 465 junior doctors, all of whom were members of the Athens Medical Association, from 14 September to 14 October 2022. Participants completed a questionnaire assessing the perceived impact of the pandemic on their training, the effectiveness of online education, and potential consequences for clinical preparedness. Factor analysis was conducted to identify underlying patterns related to perceptions for the impact on medical training. Multiple linear regression models were used to assess potential associations among the extracted factors and participants’ sociodemographic characteristics. **Results:** Among the 465 participants, the mean age was 32.1 (SD = 7.0) years and 300 (64.5%) were female. Among the responders, the majority (*n* = 241, 51.8%) conducted training in Internal Medicine, 155 (33.3%) in a surgical specialty and 69 (14.8%) in other specialties, including Psychiatry, Radiology and Laboratory Medicine. Two out of five medical students reported that their medical training was mostly affected during the first wave of the pandemic, from March to June 2020 (*n* = 201, 43.2%). Factor analysis revealed the existence of two factors with high reliability and acceptable validity, interpreted as “perceptions towards online training” and “perceptions for the consequences of the pandemic on medical training”. Age and medical specialty were found to be significantly associated with both factors. **Conclusion:** Training was severely disrupted, with potential long-term implications for clinical competence; therefore Government and Universities should consider the lessons learned from the pandemic and compensate for the time and opportunities lost. Measures must be taken to safeguard medical education and training in the event of such outbreaks in the future.

## 1. Introduction

In December 2019, a novel infection with pneumonia-like symptoms, later named Coronavirus Disease 2019 (COVID-19), was first identified in Wuhan, Hubei Province, China. The virus spread swiftly worldwide, causing significant disruptions across China and many other countries. On 11 March 2020, the World Health Organization (WHO) [1] declared it a global pandemic. As of August 2023, WHO reports [2] that there have been over 769 million confirmed cases of COVID-19 globally, with a total of 6,955,141 related deaths.

The COVID-19 pandemic caused an unprecedented disruption to both medical education and healthcare systems worldwide [3,4,5,6]. Due to the virus’s highly contagious nature, continuing traditional lectures became challenging, which significantly impacted the medical education model, traditionally built on in-person lectures and patient-centered learning. The pandemic posed serious risks to health, creating substantial obstacles for medical training. Instructors were faced with the challenge of delivering lectures in a safe manner, while maintaining the continuity and quality of education. These difficulties were compounded by limited patient care, as hospitals and clinics focused primarily on COVID-19 cases, which reduced the availability of bedside teaching for medical students and junior doctors. As a result, many were unable to complete their clerkships [3]. Clinical rotations were halted, and elective surgeries were postponed. Additional concerns included the risk of medical students contracting and spreading the virus, as well as the necessity for students to stay at home and adhere to social distancing protocols [7]. Given these challenges, there is a pressing need to develop a medical curriculum that supports ongoing learning for trainees while minimizing delays caused by the pandemic [8]. The pandemic also affected clinical practice, particularly the management of major diseases, as healthcare systems had to rapidly adapt to remote and technology-assisted approaches. Telemedicine and digital health tools played a critical role in ensuring continuity of care for patients with chronic illnesses, while minimizing in-person interactions to reduce infection risk [9].

Prior to the COVID-19 pandemic, medical training in Greece was predominantly delivered through traditional classroom settings that relied on didactic lecture formats and hands-on clinical rotations, which allowed for direct patient interaction and in-person mentorship. However, as the pandemic disrupted these conventional methods, educational institutions rapidly shifted towards digital platforms. Most proposed methods included online video lectures, interactive discussions, as well as the use of various programs or pre-recorded self-study lectures made available online for medical students. These resources were developed and provided by universities ensuring continuity in training despite the limitations imposed by the pandemic. However, educators had also to continue delivering medical education and patient care throughout the pandemic, ensuring that these services aligned with ethical principles grounded in beneficence, and the professional virtues of courage and self-sacrifice. In response to the suspension of clinical clerkship rotations, virtual clinical experiences were proposed as an alternative. This approach would allow medical residents to interview patients, collaborate with supervisors on treatment plans, assist with administrative tasks, and provide counseling on illness and prognosis [4].

In Greece, two significant lockdowns were imposed that impacted public gatherings and educational activities. The first lockdown began on 23 March 2020, with restrictions gradually being lifted by 4 May 2020. A second lockdown was implemented on 5 November 2020, and lasted until 18 January 2021. During the pandemic, all medical school lectures were suspended, forcing students to remain at home. Elective operations and procedures were canceled, and most healthcare resources were redirected to combat the COVID-19 crisis. This disruption severely affected the training of junior doctors, prompting a shift to online teaching for ongoing professional development. However, technical issues such as blackouts and poor internet connectivity posed challenges for these online platforms. As some departments began offering online lectures for medical residents, it became essential to assess the feasibility and effectiveness of this approach in supporting junior doctors’ continued education.

The primary objective of this study is to assess the perceived impact of the COVID-19 pandemic on medical training among junior doctors in Greece. The secondary objectives are to evaluate the effectiveness of online education as an alternative to traditional in-person training, examine the associations between sociodemographic factors and perceptions of training disruptions, and explore the potential long-term implications of these disruptions on clinical education.

## 2. Methods

This study follows the Strengthening the Reporting of Observational Studies in Epidemiology (STROBE) guidelines for observational research [10]. We conducted a descriptive, cross-sectional online survey from 14th September 2022 to 14th October 2022. The survey involved a questionnaire sent by email to trainees in Greece of various specialties among the members of the Athens Medical Association (A.M.A.), which is the largest medical association in Greece. The selection criteria involved membership to A.M.A. and trainee status. The survey targeted only trainees working in Greek National Health System hospitals, excluding those in private and military hospitals. A structured, anonymous short questionnaire was used. The study was conducted according to the principles of the Declaration of Helsinki of 1975, as revised in 2008. The participants provided anonymous informed consent on the survey platform before they could proceed to the electronic completion of the questionnaire. The protocol of the study was approved by the Board of the A.M.A. (Code: 30580/30.8.2022; date of approval 30 August 2022).

The study questionnaire was distributed to trainees across all specialties via their personal email addresses, with follow-up messages sent to ensure proper participant selection. A polite reminder was also sent to encourage participation and maximize the response rate. Once completed, the questionnaires were collected and the data was organized in an Excel spreadsheet for analysis. To minimize potential bias, participants were kept unaware of the study’s purpose and outcomes. The questionnaire was self-administered, with no input from the authors or other individuals, and did not collect any identifying information to maintain participant confidentiality.

### 2.1. Study Size

The sample size was determined using the OpenEpi platform with a 97% confidence level and a 5% margin of error [11]. Given that the Athens Medical Association (A.M.A.) has approximately 3000 active members, the minimum required sample size was calculated to be 408 participants.

### 2.2. The Questionnaire Format

The survey instrument was developed to assess the perceived impact of the COVID-19 pandemic on medical training, with a focus on both the effectiveness of online education and broader training challenges. Inspired by previous studies which examined the impact of the pandemic on medical education and online learning experiences, we adjusted our instrument to capture the unique situation in Greece [3,4,5]. The survey instrument was a structured, anonymous online questionnaire that explored participants’ perceptions of the impact of the pandemic on their medical training, that had three parts.

The first part included four questions on participants’ sociodemographics (sex, age, specialty, time of training), and the second part included 12 questions on participants’ perceptions about the impact of COVID-19 pandemic on medical training.

The third part included two questions about potential ending of medical training due to the pandemic. In more detail, the second part included two general questions and 10 questions related to the difficulty of online training, to its usefulness, to the general impact of the pandemic on medical training, and to potential psychological effects. The two general questions were stated in the form of a 5-point Likert scale with values labelled as 1 = “not at all”, 2 = “only a little”, 3 = “neutral”, 4 = “much”, and 5 = “very much”. The 10 questions were also stated in the form of a 5-point Likert scale but with the following labelling: 1 = “totally disagree”, 2 = “disagree”, 3 = “neither agree nor disagree”, 4 = “agree”, and 5 = “totally agree”. Six questions (Q2–Q7) out of 12 had an opposite wording (reversed-coded questions), therefore the scores on these questions were reversed (i.e., from “very much” (1) to “not at all” (5) and from “totally agree” (1) to “totally disagree” (5)). According to Pimentel, the five-point Likert scale is considered an interval scale. The mean is very significant. The range from 1 to 1.80 is interpreted as strongly disagree; 1.81 to 2.60 as disagree; 2.61 to 3.40 as neither agree nor disagree; 3.41 to 4.20 as agree and 4.21 to 5 as strongly agree [12]. This approach was used for the interpretation of the mean value of the factors. For factors consisting only of from reversed-coded questions, the range from 1 to 1.80 was interpreted as strongly agree; 1.81 to 2.60 as agree; 2.61 to 3.40 as neither agree nor disagree; 3.41 to 4.20 as disagree and 4.21 to 5 as strongly disagree. The full questionnaire is available upon reasonable request.

### 2.3. Statistical Analysis

#### 2.3.1. Factor Analysis and Reliability and Validity Assessment

Factor analysis was performed only on the questions (items) of the second part of the questionnaire and specifically, on 10 out of 12 items. Q1 and Q2 were disregarded since they were general questions stated with different options than the rest of the items. We conducted factor analysis to investigate for the existence of underlying factors in our data. In that case, the number of variables could be reduced into a smaller set of factors, where each factor represents a group of highly interrelated variables. We used the Kaiser-Meyer-Olkin (KMO) measure of sampling adequacy and the Bartlett’s test of Sphericity to check for the data appropriateness for this kind of analysis. We selected the principal components method based on the covariance matrix and the promax rotation method with Kaiser normalization. Factors were identified among the intercorrelations of a group of variables. To determine the number of factors to retain, we used the eigenvalue rule, the Scree plot, the cumulative percent of variance explained, and interpretability criteria. Moreover, all the retained factors had to consist of ≥3 items with a factor loading >0.40. Items that best defined each factor were considered those with the highest absolute factor loadings. We also calculated the ratio of factor loadings to check for potential cross-loading.

Reliability and convergent validity of the factors was assessed by the Cronbach’s alpha coefficient (α) and the corrected item-total correlation, respectively. Factors’ discriminant validity was evaluated by comparing the correlation between the factors with the factors’ alpha coefficients. Spearman’s correlation coefficient was used to measure the strength and direction of the correlation between the factors [13,14,15,16,17,18].

#### 2.3.2. Association of Factors and Sociodemographic Characteristics

For descriptive purposes, the categorical variables were presented using absolute and relative frequencies and the continuous variables using mean and standard deviation (SD). Multiple linear regression analysis was carried out separately for each extracted factor to assess potential association with participants’ sociodemographic characteristics. In the context of regression analysis, a new variable was generated by adding the scores of the items defining the factor and was included in the model as dependent variable, while age, sex, medical specialty, and time of training were included as independent variables.

The *p*-value for statistical significance was set to 0.05. SPSS 23.0 and Stata 14.2 statistical packages were used for the statistical analyses [19,20].

## 3. Results

The total number of the participants was 465 junior doctors, all of whom were members of A.M.A. Among them, 300 (64.5%) were female and 165 (35.5%) were male. The mean age was 32.1 years (SD = 7.0). 241 of the responders (51,8%) were training in Internal Medicine, 155 (33.4%) in a surgical specialty and 69 (14.8%) in other specialties, including Psychiatry, Radiology and Laboratory Medicine. Almost ¼ (*n* = 120) of the medical trainees were in the 4th year of studies, 11.4% (*n* = 53) in the 1st year, 22.6% (*n* = 105) in the 2nd year, 21.1% (*n* = 98) in the 3rd year, 9.6% (*n* = 45) in the 5th year and 9.5% (*n* = 44) in the 6th year of training, respectively (Table 1).

### 3.1. Impact of the Pandemic on Medical Training

The majority of participants (43.2%) reported that the first wave of the pandemic (March to June 2020) had the most significant impact on their training. Only a small fraction (3.2%) felt that the fifth wave of COVID-19, starting in May 2023, had influenced their training. A considerable number of respondents (26.2%) expressed their intent to stop or had already stopped their medical training due to the pandemic, with reasons including changing specialty training (28.7%), restarting specialty training in a hospital abroad (27.9%), restarting specialty training after the end of the pandemic (27%), and changing hospitals (16.4%).

In terms of satisfaction with online education, only 30.7% of the participants expressed high or moderate levels of satisfaction. Conversely, 17.6% reported being not at all satisfied. Regarding the effectiveness of online classes, 54.8% agreed that they were effective. When participants were asked about adapting to the new learning methods, 61.3% found it easy and quick. However, only 14.4% of the participants agreed that online education provides the same participatory opportunities as traditional in-person education. Regarding the continuity of online education post-pandemic, 53.1% were in favor. The vast majority of the participants (91.8%) agreed that the pandemic has an impact on their medical skills development. Also, 95.0% believed that the pandemic would have a lasting impact on their educational experience.

### 3.2. Factor Analysis and Factors’ Reliability and Validity

The KMO measure and the Bartlett’s test of Sphericity verified the appropriateness of the study data for factor analysis. Specifically, the KMO value was 0.903 (>0.50) and the *p*-value of the Bartlett’s test of Sphericity was <0.001. All 10 items were retained since all estimated communalities were >0.30. The results from factor analysis are presented in Table 2. Analysis revealed the existence of two underlying factors in our data interpreted as “Perceptions for the consequences of the pandemic on medical training” (factor 1 including 5 items: Q8–Q12) and “Perceptions towards online training” (factor 2 including 5 items: Q3–Q7). The total variance explained by both factors was 78.5% (62.9% for factor 1 and 15.6% for factor 2) and the eigenvalues of the factors were well above 1.0. The Scree plot and the interpretability criteria that were set also highlighted the retention of these two factors likewise. All items for both factors were considered stable since their factor loadings were >0.40 (pattern matrix). There was no cross-loading in any of the items (ratio of factor loadings <75%). All the item-total and corrected item-total correlations were >0.30 indicating that each item had a contribution to the total score.

The reliability was assessed over all the items of the questionnaire and also separately for the items of each factor and the results are represented in Table 3. The Cronbach’s alpha coefficient for all items was 0.931, for factor 1 reflecting physicians’ perceptions for the consequences of the pandemic on medical training was 0.931, and for factor 2 reflecting perceptions towards online training was 0.913. In all cases, the alpha coefficient was well above the recommended value of 0.60 indicating a very high reliability. In addition, the exclusion of any of the items for each factor did not result in a considerable improvement of the factor’s alpha coefficient. Regarding the factors’ convergent validity, it was considered acceptable since the corrected item-total correlation of all items for each factor was >0.50. The discriminant validity was considered adequate since the Spearman’s correlation coefficient indicating the correlation between the two factors was 0.69 (*p*-value < 0.001) which was lower than the factors’ alpha coefficients (i.e., 0.931 and 0.913).

The mean score for factor 1 reflecting physicians’ perceptions for the consequences of the pandemic on medical training was 3.9 (SD = 0.9) indicating that participants tend to agree on the significant impact of the pandemic. The mean score for factor 2 reflecting physicians’ perceptions towards online training was 2.8 (SD = 0.9) indicating a neutral perception of the participants regarding the usefulness of online training.

### 3.3. Factors and Sociodemographic Characteristics Association

Results from the multiple linear regression analyses of the association between sociodemographic characteristics and the extracted factors are presented in Table 4. Controlling for potential confounders, age and medical specialty were associated with both factors (*p* < 0.001). An increase in age was associated with a higher score in both factors. Furthermore, physicians who performed their medical training in a Surgical Department or other departments scored on average higher in both factors than those who trained in an Internal Medicine Department. Finally, female doctors scored on average higher than males in factor 1 reflecting physicians’ perceptions for the consequences of the pandemic on medical training; however, this finding was on the borderline of significance (*p* = 0.05).

## 4. Discussion

The COVID-19 pandemic led to severe disruption in training across most specialties and progression of junior doctors. This period coincides with the initial outbreak and the implementation of strict lockdown measures, which drastically altered the clinical training environment. The reduction in patient interactions and the shift to online training modalities were primary factors contributing to the negative perceptions. These findings align with previous studies that have documented the disruptive effects of the pandemic on medical education globally [21,22,23]. A lot of face-to-face activities were suspended; Universities and Hospitals had to transfer all the educational programs online in a very short period of time [24]. Transitioning to online teaching was a challenging task [25]. Although the curriculum remained the same, the way content was delivered changed. Existing content used for face-to-face lectures had to be adjusted so that to fit for an online platform and therefore optimize the experience received by the students and trainees [21].

Concerns have also been raised by educators, students and parents of undergraduates about how it is possible that programmes can reach their goals online when compared to those on campus [26]. Concerns regarding the social wellbeing of students were also raised. As highlighted by A. Fazackerley et al. and S. Thompson et al., online teaching could prove isolating, or students could be left to fend for themselves [22,27].

On the other hand, online teaching can be an enjoyable experience for students and effective [28]. However, preparation of online platforms and adaptation of content by tutors is required in order to provide trainees with a great experience as noted by T. Fawns et al. [21]. There were a lot of challenges during the COVID-19 outbreak, but the fact that there has been a significant investment into online platforms and online delivery of teaching should make us feel optimistic that online content can play an adjuvant role in medical training in the years to come.

This study, involving 465 junior doctors members of the A.M.A., provides a comprehensive assessment of the perceived impact of the COVID-19 pandemic on medical training in Greece, with a focus on the experiences of junior doctors across multiple specialties. The findings highlight the significant challenges posed by the abrupt transition to online education and the broader consequences of the pandemic on medical training and professional development. Specifically, the study results describe the significant impact of the COVID-19 pandemic on medical trainees’ training, well-being, and career choices. The findings indicate that a majority of junior doctors (51.8%) were pursuing training in Internal Medicine, with others specializing in surgical fields or various other specialties. Interestingly, the study shows that the pandemic’s impact on medical training was perceived differently across various aspects of education. Physicians trained in a Surgical Department (or other departments) believed more than those who trained in an Internal Medicine Department that the pandemic had a significant negative impact on their medical training and that online training was not useful. According to multiple studies, trainees in surgical specialties felt the impact of the pandemic more compared to other specialties [3,29]. This could be explained as all elective work was suspended due to the need for Intensive Care Unit (ICU) beds for patients that needed level 3 care [30]. Surgical training inherently requires a high degree of practical, hands-on experience, which was severely limited during the pandemic, due to the cancellation of elective surgeries and reduced patient volumes. As highlighted by N. Ilonzo et al., the volume of vascular cases in 2020 compared to 2019 was reduced by 74% [23]. Surgical trainees had very few opportunities to assist in theatre and progress in their competencies as highlighted by the REINS initiative and JA Khusid et al. [31,32]. A cross-sectional study conducted with the participation of 34 countries revealed that most respondents reported significant disruption or complete discontinuation of all aspects of surgical training and the lack of continuous professional development through conferences and courses [32]. As reported by E. Abati et al., most surgical trainees felt their competencies were reduced as a result of the pandemic. Conversely, trainees in medical specialties felt that their abilities improved during the pandemic [3].

We found that the age of physicians had an impact on their perceptions. Older trainees reported a more negative impact of the pandemic on their training and also they believe that online training was not useful. This may be due to their advanced stage in training, where clinical exposure and hands-on experience are necessary. Grantcharov T.P. et al. emphasize that direct practice is essential in residency to achieve procedural competency, which simulations alone cannot fully replicate. The authors argue that structured, real-patient interactions improve skill retention and clinical outcomes, underscoring the irreplaceable value of hands-on training in medical education [33]. Additionally, the increased responsibilities and expectations at senior levels could have exacerbated the perceived disruption caused by the pandemic.

Interestingly, it was revealed that female doctors experienced more negative impacts from the pandemic on their medical education. Although this finding was on the borderline of significance, it suggests that sex-related differences in medical training experiences may exist, potentially related to increased responsibilities or stressors outside of work during the pandemic. A study in Vienna indicated that female healthcare professionals experienced particularly high levels of stress and burnout during the COVID-19 pandemic, often due to structural challenges and increased workloads, which were amplified in certain hospital settings. These pressures affected women’s work experiences uniquely, potentially due to greater responsibilities outside of work and differing support structures compared to their male counterparts [34].

While 61.3% of participants reported that they managed to adapt quickly to online education, this adaptation was viewed negatively in the context of training and professional development. Online training may introduce significant barriers to effective learning [35]. The lack of direct, face-to-face interaction with patients and instructors, combined with technical difficulties and the possibility of distractions, could contribute to a perception that online education was not an adequate substitute for in-person training. Additionally, the limited opportunities for hands-on training could be a critical shortcoming, particularly for specialties that rely on procedural experience [36].

Furthermore, the pandemic’s impact from the beginning was explored, with the first wave in 2020 being the most challenging period for junior doctors. However, it is considerable that a small but notable group of respondents reported that the fifth wave in 2023 had influenced their medical training. This suggests that the effects of the pandemic are ongoing and evolving, making it crucial for medical institutions to remain adaptable in their educational approaches. Also, the high number of positive cases being examined, and the burden of everyday work could be another reason to explain these results. A qualitative study in Scotland highlighted that junior doctors reported substantial disruptions to their training due to the pandemic, with a notable proportion experiencing burnout and increased stress related to their work conditions and educational adjustments [37].

Finally, the study recorded a substantial proportion of junior doctors either stopped or planned to stop medical training due to the pandemic. A notable percentage of 26.2% of respondents had either considered or decided to discontinue their training due to the pandemic. The primary reasons included changing specialties, restarting training abroad, and waiting until the end of the pandemic to resume their education. This highlights a critical issue for medical workforce planning and the need for supportive measures to retain trainees within the healthcare system. Reports from studies on junior doctors during COVID-19 show substantial impacts on their mental health and training progression, suggesting that better planning for future crises is essential [38]. Institutions and policymakers should find ways to provide support and guidance to junior doctors facing career uncertainties in the wake of the pandemic. A similar study in UK highlights the need for workforce retention strategies and the importance of flexible training structures to accommodate junior doctors’ needs [39].

In addition to the challenges faced in medical training and the results observed in nursing education, our findings underscore the growing importance of virtual education modalities. Recent evidence supports the notion that simulation-based training can effectively bridge the gap between theoretical learning and practical skill development. Notably, a study demonstrated that simulation environments provide a safe, controlled setting for trainees to enhance both patient safety and clinical preparedness [40]. This approach is particularly relevant in the context of the COVID-19 pandemic, where traditional hands-on training has been significantly disrupted. The COVID-19 pandemic caused severe impact on social life and work satisfaction as highlighted by E. Abati et al. [3]. This study also analyzed the psychological effects of the pandemic on junior doctors. The disruption of routine, increased workload due to COVID-19 patient care, and the stress of adapting to new training modules contributed to a significant barrier on mental health and well-being. It is essential to acknowledge the unique challenges faced by trainees in maintaining their mental well-being during these trying times [41]. According to Di Lorenzo et al., in Italy a type of mental health support was provided to both patients with chronic conditions and to trainees looking after them in order to maintain their mental wellbeing whereas in the United States of America according to JA Khusid et al., urology residents were stressed having been redeployed in highly stressful, unfamiliar clinical settings while caring for COVID-19 patients [31,42].

## 5. Limitations

Some limitations should be considered when interpreting the findings of this study. Firstly, the study’s sample was exclusively limited to trainees who are members of the A.M.A. While this provided valuable insights into the experiences of this specific group, it may not fully represent the population of junior doctors in Greece. However, it should be noted that A.M.A. covers more than two thirds of doctors in Greece. Secondly, the study relied on self-reported data, which is subject to potential response bias. Participants’ responses may have been influenced by social desirability or by emotional states at the time of the survey; this potentially affects the accuracy of the reported experiences. Thirdly, the cross-sectional design of the study limited our ability to capture changes over time. Trainees’ experiences and perceptions may have evolved as the pandemic progressed. Also, the use of a Likert scale to measure responses, while common in survey research, may have inherent limitations [9]. The interpretation of Likert scale categories can vary among participants [9]. Lastly, participants were asked to recall their experiences and perceptions during different waves of the pandemic, potentially introducing recall bias.

## 6. Conclusions

The COVID-19 pandemic has had a profound and lasting impact on medical training in Greece, particularly for specialties that require hands-on experience. While online education provided a temporary solution during the pandemic, it was largely viewed as inadequate for the training needs of junior doctors. This study highlights the need for medical institutions to develop flexible, hybrid educational models that combine the advantages of online learning with the essential elements of in-person clinical training. As the healthcare sector continues to navigate the ongoing challenges created by the pandemic, it is crucial to ensure that future medical trainees receive the comprehensive education and training they need to succeed in their professions.

## Figures and Tables

**Table 1:** Demographic characteristics of the responders.

		N	%
Sex	Male	165	35.5
	Female	300	64.5
Medical specialty	Internal Medicine Department	241	51.8
	Surgical Department	155	33.4
	Other Departments *	69	14.8
	* Including Psychiatry, Radiology and Laboratory Medicine		
Age (in years, mean ± SD)		32.1 ± 7.0	
Time of training	1st year	53	11.4
	2nd year	105	22.6
	3rd year	98	21.1
	4th year	120	25.8
	5th year	45	9.6
	6th year	44	9.5

N = Absolute Number; % = Relative frequency; SD = Standard Deviation.

**Table 2:** Descriptive statistics and factor analysis results.

Number	Question (Item)	Mean (SD)	Factor Loading		Factor
			F1	F2	
1	Has the pandemic affected your medical training?	4.3 (0.8)	Excluded from factor analysis		
2	How satisfied are you from online training?	3.3 (1.1)	Excluded from factor analysis		
3	Do the online lessons have effectiveness	2.5 (0.9)	0.004	0.636	Online training
4	I managed to adapt to online trainings easy and quick	2.5 (0.9)	0.027	0.883	Online training
5	Online and in-person training provide the same opportunities to participate	3.8 (1.0)	−0.023	0.877	Online training
6	Online training has positive impact to my medical skills	2.5 (1.1)	−0.018	0.935	Online training
7	After the pandemic online training should continue	2.6 (1.2)	0.035	0.933	Online training
8	The pandemic has affected your training in dealing with the hospital environment	3.8 (1.1)	0.920	0.009	Consequences of the pandemic
9	The pandemic affects the creation and acquisition of your medical skills that are necessary during your specialty	4.4 (0.6)	0.916	−0.058	Consequences of the pandemic
10	The pandemic will have an impact during your training in your specialty	4.5 (0.6)	0.872	−0.058	Consequences of the pandemic
11	The pandemic will have an impact on my future academic activity (completion of studies, participation in a postgraduate study program or preparation of a doctoral thesis)	3.6 (1.1)	0.915	0.041	Consequences of the pandemic
12	The pandemic has reduced my desire to engage in my specialty and medical science in general	3.2 (1.2)	0.862	0.136	Consequences of the pandemic
Total variance explained (78.5%)			62.9%	15.6%	

SD = Standard Deviation; F1 = Factor 1; F2 = Factor 2.

**Table 3:** Item analysis and reliability statistics of the factors.

Factor	Item-Total Correlation	Corrected Item-Total Correlation	Cronbach’s Alpha (If Item Deleted)	Cronbach’s Alpha (Factor)
**F2: Online training**				0.913
3. Do the online lessons have effectiveness	0.686	0.553	0.935	
4. I managed to adapt to online training easily and quickly	0.892	0.833	0.885	
5. Online and in-person training provide the same opportunities to participate	0.867	0.791	0.892	
6. Online training has a positive impact on my medical skills	0.908	0.845	0.880	
7. After the pandemic, online training should continue	0.944	0.899	0.868	
**F1: Consequences of the pandemic**				0.931
8. The pandemic has affected your training in dealing with the hospital environment	0.916	0.853	0.911	
9. The pandemic affects the creation and acquisition of your medical skills that are necessary during your specialty	0.896	0.860	0.923	
10. The pandemic will have an impact during your training in your specialty	0.859	0.810	0.928	
11. The pandemic will have an impact on my future academic activity (completion of studies, participation in a postgraduate study program or preparation of a doctoral thesis)	0.939	0.896	0.900	
12. The pandemic has reduced my desire to engage in my specialty and medical science in general	0.939	0.884	0.910	

Item total: Correlation of the item with the overall score; Corrected item-total: Correlation of the item with the overall score excluding the item. Cronbach’s alpha coefficient (α) for all items = 0.931.

**Table 4:** Multiple linear regression analysis using the factor score (sum of the scores of the items that best define the factor) as the outcome variable.

F1: Consequences of the Pandemic			
Explanatory Variable	Coefficient	95% Conf. Interval	*p*-Value
Age (years)	0.24	(0.19, 0.29)	<0.001
Sex			
Male	Reference category		
Female	0.68	(−0.0003, 1.37)	0.050
Medical specialty			
Internal Medicine Department	Reference category		
Surgical Department	2.24	(1.47, 3.02)	<0.001
Other Departments *	2.05	(1.04, 3.05)	<0.001
Time of training			
1st year	Reference category		
2nd year	0.28	(−0.92, 1.47)	0.647
3rd year	−1.28	(−2.49, −0.07)	0.038
4th year	0.14	(−1.03, 1.30)	0.818
5th year	−0.30	(−1.73, 1.14)	0.686
6th year	−0.35	(−1.79, 1.09)	0.636
**F2: Online training**			
**Explanatory variable**	**Coefficient**	**95% Conf. Interval**	***p*-value**
Age (years)	0.35	(0.31, 0.40)	<0.001
Sex			
Male	Reference category		
Female	0.50	(−0.08, 1.09)	0.090
Medical specialty			
Internal Medicine Department	Reference category		
Surgical Department	2.96	(2.29, 3.62)	<0.001
Other Departments *	1.72	(0.87, 2.58)	<0.001
Time of training			
1st year	Reference category		
2nd year	−0.12	(−1.14, 0.90)	0.822
3rd year	−0.31	(−1.34, 0.73)	0.560
4th year	0.19	(−0.81, 1.18)	0.709
5th year	0.17	(−1.05, 1.40)	0.779
6th year	−0.22	(−1.45, 1.01)	0.728

* Including Psychiatry, Radiology and Laboratory Medicine.

## Data Availability

Data are available in a public, open access repository.
